# Impact of Bacterial Toxins in the Lungs

**DOI:** 10.3390/toxins12040223

**Published:** 2020-04-02

**Authors:** Rudolf Lucas, Yalda Hadizamani, Joyce Gonzales, Boris Gorshkov, Thomas Bodmer, Yves Berthiaume, Ueli Moehrlen, Hartmut Lode, Hanno Huwer, Martina Hudel, Mobarak Abu Mraheil, Haroldo Alfredo Flores Toque, Trinad Chakraborty, Jürg Hamacher

**Affiliations:** 1 Pharmacology and Toxicology, Medical College of Georgia at Augusta University, Augusta, GA 30912, USA; hflorestoque@augusta.edu; 2 Vascular Biology Center, Medical College of Georgia at Augusta University, Augusta, GA 30912, USA; BGORSHKOV@augusta.edu; 3 Department of Medicine and Division of Pulmonary Critical Care Medicine, Medical College of Georgia at Augusta University, Augusta, GA 30912, USA; jgonzales@augusta.edu; 4 Lungen-und Atmungsstiftung, Bern, 3012 Bern, Switzerland; y.hadizamani@gmail.com; 5 Pneumology, Clinic for General Internal Medicine, Lindenhofspital Bern, 3012 Bern, Switzerland; 6 Labormedizinisches Zentrum Dr. Risch, Waldeggstr. 37 CH-3097 Liebefeld, Switzerland; thomas.bodmer@risch.ch; 7 Department of Medicine, Faculty of Medicine, Université de Montréal, Montréal, QC H3T 1J4, Canada; yves.berthiaume@umontreal.ca; 8 Pediatric Surgery, University Children’s Hospital, Zürich, Steinwiesstrasse 75, CH-8032 Zürch, Switzerland; ueli.moehrlen@bluewin.ch; 9 Insitut für klinische Pharmakologie, Charité, Universitätsklinikum Berlin, Reichsstrasse 2, D-14052 Berlin, Germany; haloheck@zedat.fu-berlin.de; 10 Department of Cardiothoracic Surgery, Voelklingen Heart Center, 66333 Voelklingen/Saar, Germany; hhuwer@t-online.de; 11 Justus-Liebig-University, Biomedical Research Centre Seltersberg, Schubertstr. 81, 35392 Giessen, Germany; martina.hudel@mikrobio.med.uni-giessen.de (M.H.); Mobarak.Mraheil@mikrobio.med.uni-giessen.de (M.A.M.); Trinad.Chakraborty@mikrobio.med.uni-giessen.de (T.C.); 12 Medical Clinic V-Pneumology, Allergology, Intensive Care Medicine and Environmental Medicine, Faculty of Medicine, Saarland University, University Medical Centre of the Saarland, D-66421 Homburg, Germany; 13 Institute for Clinical & Experimental Surgery, Faculty of Medicine, Saarland University, D-66421 Homburg, Germany

**Keywords:** bacterial toxins, alveolar-capillary barrier, host defense, alveolar liquid clearance, inflammation, pulmonary edema

## Abstract

Bacterial toxins play a key role in the pathogenesis of lung disease. Based on their structural and functional properties, they employ various strategies to modulate lung barrier function and to impair host defense in order to promote infection. Although in general, these toxins target common cellular signaling pathways and host compartments, toxin- and cell-specific effects have also been reported. Toxins can affect resident pulmonary cells involved in alveolar fluid clearance (AFC) and barrier function through impairing vectorial Na^+^ transport and through cytoskeletal collapse, as such, destroying cell-cell adhesions. The resulting loss of alveolar-capillary barrier integrity and fluid clearance capacity will induce capillary leak and foster edema formation, which will in turn impair gas exchange and endanger the survival of the host. Toxins modulate or neutralize protective host cell mechanisms of both the innate and adaptive immunity response during chronic infection. In particular, toxins can either recruit or kill central players of the lung’s innate immune responses to pathogenic attacks, i.e., alveolar macrophages (AMs) and neutrophils. Pulmonary disorders resulting from these toxin actions include, e.g., acute lung injury (ALI), the acute respiratory syndrome (ARDS), and severe pneumonia. When acute infection converts to persistence, i.e., colonization and chronic infection, lung diseases, such as bronchitis, chronic obstructive pulmonary disease (COPD), and cystic fibrosis (CF) can arise. The aim of this review is to discuss the impact of bacterial toxins in the lungs and the resulting outcomes for pathogenesis, their roles in promoting bacterial dissemination, and bacterial survival in disease progression.

## 1. Introduction

Bacterial toxins are extremely powerful and sophisticated molecular weapons produced by Gram-negative and Gram-positive pathogens [1]. Based on their structure and function, they may exhibit diverse biological activities during infection [2]. In view of their continuous exposure to the external environment, the lungs represent a particularly vulnerable organ for infection with bacteria and their exo- and endotoxins. The lung is conceptually a blood-filled sponge that is interposed in series in the bodies’ systemic circulation to take up oxygen and remove carbon dioxide; it includes a thin epithelial alveolar layer in its alveoli that is about 100 times greater than the epithelial surface of the skin. This thin epithelial layer needs to remain intact in order to assure efficient gas exchange capacity [3]. The unique architecture of the airways, with 23 levels of branching, starting from the trachea, the bronchi, and the bronchioli all the way down to the 600–700 million alveoli, provides an impressive alveolar surface area of up to 140 m^2^ for gas exchange with an area of 80–90 m^2^ of pulmonary capillaries. In comparison, a sphere with a similar volume as the lungs (about 4 L) would provide a 1000-fold lower surface area. As indicated above, for the lungs to provide efficient gas exchange, it is crucial that the alveolar-capillary barrier keeps its integrity [3,4,5,6]. Even a small alteration in the structural or functional properties of this epithelial layer, as can be caused by bacterial toxins, can lead to the onset or exacerbation of lung disease [4,6]. Bacterial toxins use various strategies to alter the viability of the lung epithelial and capillary endothelial surface [4,6].

Classically, bacterial toxins are categorized into two groups, as exotoxins and endotoxins, respectively [7,8]. Exotoxins are single polypeptides or heteromeric protein complexes, synthesized inside either Gram-positive or Gram-negative bacteria and released by various bacterial secretion systems from bacterial colonization sites towards target cells in the alveoli or in the systemic circulation [7,8]. However, some exotoxins are unleashed following autolysis or upon antibiotics-induced bacteriolysis [9]. According to their main mechanism of action, exotoxins can be classified into three major groups, i.e., (1) membrane acting, (2) membrane damaging, and (3) intracellular effectors [10]. Membrane acting toxins bind to specific receptors on the surface of host cells and subsequently transduce transmembrane signals [10]. Membrane damaging toxins form pores or disrupt lipid bilayers to manipulate ion homeostasis, as such launching pathways involved in cell death and barrier dysfunction, like Ca^2+^ [10,11]. Intracellular effector toxins manipulate target molecule(s) by translocating an active enzymatic component into the cell [10]. All of these mechanisms may be involved in bacterial toxin-induced lung injury.

Exposure to bacterial toxins may cause direct alveolar epithelial and capillary endothelial cell death. Epithelial cells death as well as decreased barrier function increases permeability of the alveolar-capillary barriers and thus fosters the generation of pulmonary permeability edema. Moreover, increased cell death in the alveolar compartment perturbs type I and II alveolar epithelial (AT1, AT2)-mediated alveolar fluid clearance and increases the risk of disordered repair, which can lead to a fibroproliferative response as seen in the acute respiratory syndrome (ARDS) [6]. Injury to the alveolar type I cells, the major component of the alveolar barrier, is a key determinant of disease evolution of acute lung injury (ALI) and ARDS. As such, the impact of toxins on AT1 should be carefully investigated [6]. Moreover, some toxins can impair the function of ion channels on the alveolar cell surface, or can form membrane pores, both of which can contribute to ion dysregulation and the launch of molecular pathways leading to epithelial barrier dysfunction and cell death [9,12,13,14,15,16,17,18,19,20,21,22]. Bacterial toxins can also target epithelial cell cilia, respiratory mucosa, and tight junction (TJ) proteins, which are important components of the lung epithelial barrier [23,24,25,26,27,28,29]. As the degree of AT1/2 and capillary endothelial cell injury are key determinants of ALI- and ARDS, the destructive impact of toxins should be carefully considered [6]. Moreover, pore-forming toxins, such as the G-positive cytolysins pneumolysin (PLY) and listeriolysin-O (LLO), can cause ion flux dysregulation (especially Ca^2+^) and launch molecular pathways fostering epithelial and endothelial barrier dysfunction and cell death [9,11,12,13,14,15,16,17,18,19,20,21,22]. In addition, epithelial cell cilia, respiratory mucosa, and TJ proteins are other targets of bacterial toxins at the pulmonary barrier [23,24,25,26,27,28,29].

Endotoxins, such as lipopolysaccharides (LPS) are the major constituents and protective elements of the outer membrane of Gram-negative bacteria [30]. LPS is comprised of three domains, which are genetically, chemically, and functionally distinctive [31]. These domains include the lipid A, the core, and the O-antigen [32,33]. LPS has a broad range of biological activities, which can disrupt lung function and is considered a causative factor in chronic lung diseases, including chronic obstructive pulmonary disease (COPD) and cystic fibrosis (CF) [34,35]. LPS administration was also shown to induce bronchopulmonary hyperresponsiveness leading to asthma. However, in this case eosinophils are not involved, but rather neutrophils and the cytokine Tumor Necrosis Factor [36,37]. LPS moreover exerts important pathogenic roles in ALI/ARDS and pneumonia. Endotoxin can foster bacterial persistence by facilitating biofilm formation, which is especially important in genetic diseases, such as CF [38,39,40].

During infection, cells of both the innate and adaptive immune response are engaged. Alveolar macrophages (AMs) are the guards of innate immunity in the alveolar space and can be resident or recruited from the systemic circulation. AMs have developed different inflammatory strategies to attack the pathogen as well as anti-inflammatory strategies for resolution of inflammation [41]. As part of their anti-inflammatory mechanisms, AMs phagocytose apoptotic cells (efferocytosis) and thereby prevent them to release toxic components, such as pro-inflammatory cytokines, chemokines, and leukotriene C4. They moreover induce the release of anti-inflammatory and repair factors, including transforming growth factor β1 (TGF-β1), prostaglandin E_2_ (PGE_2_), and platelet-activating factor (PAF) [42,43,44,45]. Although PGE_2_ was shown to be involved in both pro- and anti-inflammatory activities, its capacity to activate endothelial nitric oxide synthase (eNOS)-mediated nitric oxide generation was shown to significantly blunt endothelial leukocyte interactions [46,47]. Moreover, mice defective in microsomal prostaglandin E synthase-1, a key enzyme in PGE_2_ synthesis, were shown to have significantly increased lethality and a defective pulmonary clearance of *Streptococcus pneumoniae* [48].

Bacterial toxins can hinder the function of AMs and neutrophils and, thereby, disturb the early innate antibacterial host immune reaction, as such facilitating a microenvironment conducive for bacterial colonization and proliferation [49,50].

Various respiratory tract disorders are associated with bacterial toxins. During the infection, diverse drivers, mediators, triggers, and catalysts contribute to infection that are coupled to feedback loops systems. Obviously, simple cause and effect paradigms are incapable of capturing these complex circumstances. Therefore, to understand such complex relationships, and in order to develop efficient antimicrobial therapies, it is essential to identify the molecular pathways that have to be targeted as well as the mechanisms mediating their dysregulation. Accordingly, without trying to cover all pathways, this review aims to summarize some current knowledge gained from published data and from our own studies on the role of bacterial toxins in the pathogenesis of acute and chronic lung diseases, both of which can have a profound impact on the life quality of individual patients and their relatives. We will moreover briefly discuss the impact of pathogenic bacteria and their toxins in lung disease pathogenesis.

## 2. *Staphylococcus aureus* (*S. aureus*)

*S. aureus* is a spherical Gram-positive aerobic opportunistic pathogen [51] with a diameter of about 0.5 to 1.0 μm [52], which often forms clusters [53]. It is a ubiquitous microorganism commonly found in normal human flora, such as skin, nasal passage, axillae, and repository tracts, but it is also the causative agent of blisters, food poisoning, and pulmonary infection [54,55,56,57,58,59,60,61]. The commonly observed *S. aureus* infection in CF patients is of high clinical importance and usually occurs before *Pseudomonas aeruginosa* infection. This represents one of the main causes of the recurrent acute or persistent pulmonary infections and progressive decline in lung function characteristic for the genetic life-threatening CF multisystem disorder. Pulmonary infections due to *S. aureus* can also occur in the community or hospital setting among individuals with *S. aureus* colonization of the skin or of the nares, particularly in the context of intubation. *S. aureus* pneumonia may occur after viral pneumonia, or typically during right-sided *S. aureus* endocarditis with septic pulmonary emboli.

Due to its colonization and virulence properties, *S. aureus* is able to cause community- and hospital-acquired infectious diseases [61]. The pathogen induces host-damaging responses by means of surface-located protein factors, polysaccharides and secreted virulence factors [62,63]. The highly regulated toxin production system of *S. aureus* is relevant to human disease [64]. In the following paragraphs, we will discuss the contribution of the *S. aureus*-derived toxins alpha-hemolysin/alpha-toxin, beta-toxin, and Panton–Valentine leukocidin to lung injury.

### 2.1. Alpha-Hemolysin (Hla)

Hla is a small β-barrel archetypal pore-forming toxin with a molecular mass of 34 kDa expressed by most strains of *S. aureus* as a water-soluble monomer [12,65]. Hla contributes to the pathogenesis of ventilator-associated pneumonia [64,66,67] through forming pores, manipulating structural and functional properties of alveolar epithelium, capillary endothelium, and AMs, and provoking inflammatory mediator release [67,68,69,70]. Pore formation occurs upon binding of Hla to its receptor A Disintegrin and Metalloprotease 10 (ADAM-10) in the target cell membrane, which induces oligomerization, self-assembly, and the generation of a lipid-bilayer mushroom-shaped hexameric/heptameric channel [67]. In comparison to other pore-forming toxins, such as pneumolysin (PLY) and listeriolysin-O (LLO), Hla makes smaller pores with a diameter of only about 1–2 nm that are permeable for Ca^2+^, Na^+^, K^+^, Cl^−^, ATP, and molecules with low molecular weight (between 1 and 4 kDa) [12,71]. Like LLO and PLY, Hla fosters transmembrane influx of Ca^2+^, which ultimately leads to the development of pulmonary edema [12,20]. Binding of Hla to ADAM10 causes activation and upregulation of its metalloprotease activity leading to the pathologic cleavage of its substrates, including epithelial E-cadherin and vascular endothelial (VE)-cadherin, with concomitant loss of barrier function [70,71,72,73,74,75,76]. Moreover, Hla has the ability to disrupt endothelial-cell TJs through activating acid sphingomyelinase and release of ceramide [69]. Ceramide produced by acid sphingomyelinase is associated with PAF-induced pulmonary edema [72].

Alveolar epithelial cells represent sensitive targets to an Hla assault [73], since the pore-forming toxin impairs the alveolar-capillary barrier of the lung in a rat model of *S. aureus*-induced pneumonia [74,75], upon inducing alterations in cell shape and by promoting formation of paracellular gaps in human airway epithelial cells [75,76]. Hla pore formation in bronchial epithelium causes the release of cytosolic ATP to the extracellular space [76,77], which increases ciliary beat frequency in tracheal cells via both P2X and P2Y receptors [78]. Hla, moreover, induces mucus secretion in goblet cells [79,80] and fosters Interleukin 6 (IL-6) secretion by small human airway epithelial cells [81]. In addition, Hla damages the function of rabbit AMs in vitro by reducing their phagocytic activity [82]. Hla, also, contributes to lung and liver injury by preventing adequate platelet repair and exacerbating the host inflammatory response, leading to the release of proinflammatory cytokines such as IL-1β, IL-6, tumor necrosis factor (TNF), Interleukin 18 (IL-18), and chemokines such as Interleukin 8 (IL-8/CXCL8) and macrophage inflammatory protein 2 (MIP-2) [82,83,84,85,86,87]. Furthermore, it has been shown in a murine model of severe pneumonia that the pore-forming activity of Hla potentiates bacterial virulence via activation of the NOD-, LRR- and pyrin domain-containing protein 3 (NLRP3) inflammasome in AMs, which stimulates the production of IL-1β and IL-18 and induces necroptosis [86,87].

### 2.2. Beta-Hemolysin (Hlb)

Hlb/beta-toxin (β-toxin) is a Mg^2+^ -dependent neutral sphingomyelinase and cytolysin, with a molecular weight of 35 kDa, secreted by certain strains of *S. aureus* [88,89]. Hlb can cause lung injury by means of enhancing neutrophil infiltration in a syndecan-1 dependent manner, accompanied by leakage of serum proteins into the lung tissue and exudation of proteins into the airways [89]. In vitro Hlb can decrease or halt ciliary beat frequency in rabbit respiratory epithelium dependent on its concentration and exposure period [27].

### 2.3. Panton-Valentine Leukocidin (PVL)

PVL is a non-hemolytic leukocytolytic exotoxin expressed by many methicillin-resistant *S. aureus* strains [90,91]. It is a two-component (32 and 34 kDa) pore-forming toxin with membrane-disturbing and cytolytic capabilities [92]. PVL-producing strains can cause severe lung necrosis, alveolar hemorrhage, pulmonary edema formation, lung hemoptysis, and sometimes death, which are all characteristics of human necrotizing pneumonia [93]. PVL is cytotoxic towards the lung epithelium, as it induces necrosis and/or apoptosis of AT1 cells [28]. PVL induces lung inflammation leading to acute lung injury in a rabbit model of necrotizing pneumonia, fostering infiltration of AMs and polymorphonuclear neutrophils (PMNs) through the release of chemokines such as monocyte chemotactic protein 1 (MCP-1) and IL-8/CXCL8 (a potent neutrophil attractant) [93,94,95,96]. Following PMN migration into the alveolar space, PVL induces PMN lysis, which leads to release of granule contents, such as proteases and reactive oxygen metabolites. Proteases together with oxidants can cause cell death of alveolar type 1 cells and perforation of the alveolar epithelial-interstitial and interstitial-endothelial barrier, providing a path for the influx of fluid and proteins from the vascular space into the alveolus; thus, ending up with pulmonary edema formation and ALI [97,98,99,100]. PVL-mediated pore formation induces influx of Ca^2+,^ in PMNs, which in turn increases intracellular Ca^2+^ levels [93]. Depending on the concentration of PVL, increased intracellular Ca^2+^ can either induce PMN degranulation, apoptosis, or -at the highest doses- octameric pore-mediated necrosis [93,95]. The size of the pore formed by PVL in PMNs is dependent upon ionic environmental conditions [93].

## 3. *Pseudomonas aeruginosa*

The Gram-negative bacterium *P. aeruginosa* is an opportunistic pathogenic bacterium [101] that normally inhabits soil and surfaces in aqueous environments [101]. *P. aeruginosa* is highly adapted to human hosts, has a moderate predilection to immunocompromised hosts, and can cause acute infections of different tissues and organs, including lungs, as is the case with ventilator-associated pneumonia, where it is the most common multi-drug resistant bacterium and probably the most serious etiologic agent [2]. Individual strains often produce a considerable number of toxins and surface components that associate with virulence properties.

*P. aeruginosa* causes persistent colonization and infection in CF patients and it is the most frequent infective agent leading to death in CF [102]. The large genome of *P. aeruginosa* encodes a wide range of different metabolic enzymes, which confers the bacterium with high nutritional versatility [102]. Counter-intuitive to the concept that bacteria cause disease by expressing their virulence factors, CF colonization is promoted by downregulation of virulence factors. The immunologic response to such virulence factors within colonizing strains promotes the emergence of mutated variants that are better at evading pulmonary host immune mechanisms. Thus, the *P. aeruginosa* flagellin synthesis is frequently downregulated in the CF lung, most probably to avoid detection by host defense mechanisms. Flagellin is highly immunogenic and may be detected by pattern recognition receptors [103]. Furthermore, virulence factors that are no longer needed for chronic colonization accumulate random mutations in the genes responsible for their expression in strains that subsequently colonize the airways.

The persistence in the face of intact host defense mechanisms early in the life of many patients with CF is a characteristic of colonizing bacteria. Thus, defects in mucociliary clearance and in host killing of bacteria in CF promote their persistence [104]. In CF, the lack of the expression of the cystic fibrosis transmembrane conductance regulator (CFTR) gene causes depletion of the periciliary liquid layer at the surface of the tracheal epithelium, which and inhibits clearance of the mucus coat from the lung, bronchial tree, and trachea [105]. With the lack of clearance of the mucus coat, bacteria that live in the mucus may colonize the tracheobronchial tree and lung and the patient ultimately suffers from lung infection.

Isolates from CF Patients with newly identified *P. aeruginosa* infection frequently have mucoid-and biofilm-forming phenotypes, which are generally associated with adaptation towards chronic persistence in the CF lung [104]. Important steps include the downregulation of the production of toxins, flagellum and pili, the loss of lipopolysaccharide “O” side chains, and the expression of the polysaccharide alginate giving the bacterium a “mucoid phenotype” promoting biofilm formation, thereby protecting the bacterium against phagocytosis and promotes tissue damage [104].

CF patients lack effective mechanisms for killing *P. aeruginosa* and are therefore more susceptible to this pathogen than others [106]. The high chloride concentrations in pulmonary secretion due to the defective CFTR is critical because this high chloride environment inactivates the secreted antimicrobial respiratory epithelial peptides beta-defensin-1 and -2 [107,108,109,110], increases inflammatory mediators by polymorphonuclear neutrophils and diminishes the ability of neutrophils to kill *P. aeruginosa* [110]. Ivacaftor, which restores CFTR function, was shown to reduce *P. aeruginosa* culture positivity in CF patients [111]. The following paragraphs will discuss the major virulence factors of *P. aeruginosa.*

### 3.1. Exotoxin A (P-ExA)

P-ExA is a 66 kDa exotoxin [112] secreted by the type II secretion system (T2SS) of the *P. aeruginosa* strain PA 103 [112]. P-ExA is a membrane-damaging toxin that can form both pH- and temperature-dependent α-helixes pores, leading to the generation of pores of about 2.8 nm in diameter [113,114,115]. It has been reported that during respiratory tract infections with *P. aeruginosa*, P-ExA can facilitate both the growth of *P. aeruginosa* [116] and promote cell infiltration in the lungs [117]. It is a weak inducer of IL-1- β, IL-6, MIP-1α and MIP-2, but does not induce TNF [118] and is an inhibitor of lung IL-18 secretion [119]. To disrupt pulmonary barrier function, P-ExA was shown to increase paracellular permeability in AT2 cells (by preventing the repair of damaged tight junctions [26]) and in the bovine pulmonary artery endothelial cells [120].

### 3.2. Exoenzyme S (Exo S) and Exoenzyme T (Exo T)

Exo S and Exo T are bi-functional type-III cytotoxins of *Pseudomonas aeruginosa* that share 76% primary amino acid homology and contain N-terminal Rho GTPase-activating proteins (GAP) domains and C-terminal adenosine diphosphate (ADP)-ribosylation domains [121,122]. The Rho GAP activities of Exo S and Exo T appear to be biochemically and biologically identical, targeting Rho, Rac, and Cdc42. Expression of the Rho GAP domain in mammalian cells results in the disruption of the actin cytoskeleton and interference of phagocytosis. Expression of the ADP-ribosyltransferase domain of Exo S elicits a cytotoxic phenotype in cultured cells, while the expression of Exo T appears to interfere with host cell phagocytic activity.

Exo S is a 49 kDa-secreted cytotoxin [121,122] with an enzymatic activity that is translocated by its type III secretion system (T3SS) into the cytosol of eukaryotic cells [29,123]. Whereas *P. aeruginosa* type III secretion performs a dominant role in acute infections, emerging evidence suggests that it may actually be selected against in chronic infections, such as those afflicting individuals with CF. Many CF patients have antibodies against type III effector proteins [124], suggesting that these factors are expressed at some point during infection. However, *P. aeruginosa* strains gradually lose the ability to secrete type III proteins over time in the CF airways [125].

It has been reported that Exo S can cause severe pulmonary-vascular breakdown [29], bacterial persistence, and progression of pneumonia [29,126]. Exo S causes disruption of bronchial epithelium cells and AT1 [29]. It damages the membrane of AT1, disrupts TJs, and causes cell death [29,127]. By annihilating focal adhesions, retraction of endothelial cells, and a rupture of the endothelial monolayer, Exo S modulates lung endothelial permeability [128,129]. During initial phases of pneumonia, *P. aeruginosa* injects Exo S into leukocytes such as neutrophils and at later time points disrupts AT1 thereby facilitating dissemination of *P. aeruginosa* from the lung to the bloodstream [29]. In addition, Exo S targets substrates of host cells, which are essential for preservation and viability of AT1 tight junctions [29]. Exo S can also cause alteration of pulmonary structure [129]. Intratracheal instillation of purified Exo S into rat lungs causes membrane damage of AT1 cells by dislodging membranes, accumulation of inflammatory cells, fibrinous exudation, and destruction of the bronchial epithelial layer [129].

In a mouse model of pneumonia, Exo S can cause the progression of inflammation leading to neutrophil infiltration into the lung [130]. Moreover, during early pneumonia, Exo S is injected into recruited neutrophils and into type I pneumocytes at later time points [29]. Exo S inhibits the phagocytic capacity of macrophages and neutrophils [130]. Interestingly, the injection of Exo S in type I pneumocytes causes an increase in their size during pneumonia [29]. In addition, by activating TLR2 and TLR4, Exo S induces a range of inflammatory responses [131]. 

Exo T is also secreted through the T3SS of *P. aeruginosa* [132]. Exo T inhibits lung epithelial wound repair by targeting cells at the edge of the wound in vitro and provoking a collapse of the actin cytoskeleton, which ends up to cell rounding and cell detachment [133]. To evade phagocytosis of immune effector cells, Exo T inhibits the internalization of *P. aeruginosa* by phagocytes and epithelial cells [133,134]. It also causes apoptosis of epithelial cells [134]. Similar to Exo S, Exo T ruptures the endothelial monolayer. By disrupting the pulmonary barrier function [129], Exo T causes the dissemination of bacterial and inflammatory mediators from the airspace into the systemic circulation that leads to bacteremia and sepsis. In vitro and in vivo data show that during *P. aeruginosa* induced pneumonia, Exo T increases interferon gamma (IFN-γ) production by natural killer (NK) cells in lungs [135,136,137].

### 3.3. Exotoxin U (Exo U)

Exo U is a 74 kDa phospholipase secreted by T3SS of *P. aeruginosa* directly into cytosol of targeted cells [138,139]. In contrast to, e.g., Exo S, Exo U has phospholipase A_2_ activity with broad substrate specificity [140]. Exo S and Exo U are rarely found together in one bacterial strain [141]. Despite their different enzymatic activities, Exo S also Exo U can both provoke cell death and epithelial damage, vascular hyperpermeability, platelet activation, and thrombus formation. Exo U can moreover impair phagocytosis ability of AMs and cause macrophage necrosis [142,143,144]. Exo U activates nuclear factor kappa light chain enhancer of activated B cells (NF-κB) and stimulates IL-8/CXCL8 secretion in *P. aeruginosa*-infected epithelial and endothelial cell lines [145], which can in turn augment neutrophil infiltration, but it blunts the production of IL-18 [141]. As such, both Exo U and Exo S, contribute to lung injury through the course of pneumonia, ALI, bacteremia and sepsis [142,143,144,145].

### 3.4. Exotoxin Y (Exo Y)

Exo Y is a 42 kDa adenylate cyclase secreted and translocated by the T3SS of *P. aeruginosa* to the host cell cytosol [146,147]. As a soluble cyclase, Exo Y is a soluble cyclase functionally similar to *Bacillus anthracis* edema factor and *Bordetella pertussis* adenylate cyclase toxin (cyaA) [148]. Exo Y contributes to the acute pathogenicity of *P. aeruginosa* by destruction of lung barrier integrity, decreasing secretion of different mediators, enhancing apoptotic activity, hemorrhage, formation of interstitial edema in alveolar septa and infiltration of the perivascular space with erythrocytes and neutrophilic granulocytes [149]. Exo Y disrupts the integrity of the pulmonary barrier by inducing epithelial and endothelial cell rounding upon modulating the actin cytoskeleton in the cell margins [149,150,151]. It inhibits endothelial cell proliferation and vascular repair following lung injury by targeting microtubules [151]. Following introduction of Exo Y into the host cell cytoplasm, it generates cyclic nucleotides [147] to activate protein kinases [152]. Such activation causes tau phosphorylation and microtubule breakdown [153], which leads to inter- endothelial cell gap formation, pulmonary vascular dysfunction and thereafter establishment of pulmonary edema, which occurs as a consequence of impaired alveolar-capillary barrier disruption, combined or not with reduced alveolar fluid clearance capacity [4,5,152,153]. In addition, Exo Y impairs recovery of the endothelial cell barrier, decreasing migration, proliferation, and lung repair [151]. Exo Y, somewhat paradoxically, inhibits rather than induces pro-inflammatory cytokine and chemokine production in macrophages, as such decreasing secretion of IL-1β, IL-6, TNF, and of the chemokine IL-8/CXCL8 [154,155].

### 3.5. Exolysin A (Exl A)

Exl A is a 172 kDa two-partner pore-forming toxin, secreted by an outlier family of *P. aeruginosa* strains that are devoid of T3SS and all TSS3-toxins [2,114,151,156,157,158]. It can cause devastating injuries in infected lungs by altering plasma membrane permeability, disrupting the alveolar-capillary barrier leading to pulmonary hemorrhages and promoting bacterial growth in the lungs and dissemination into the body [2,159,160]. Exl A can cause permeability in the plasma membrane of host cells by induction of plasma membrane rupture in epithelial, endothelial, and immune cells [161]. It creates pores with a diameter of about 1.6 nm by employing the type four (IV) pili for adhesion of bacteria to the target cell and facilitating direct contact between bacteria and host cell walls [114]. Release of Exl A in the local medium close to the host cell membrane leads to insertion into the host cell membrane, oligomerization, and pore formation. Like other pore-forming toxins, Exl A promotes junction disruption to facilitate the trafficking of small molecules such as Ca^2+^ and K^+^ [2,114]. Influx of Ca^2+^ into the cytosol, dissociates calmodulin from pro-ADAM10 [160], as such, allowing mature ADAM10 (m-ADAM10) to cleave the adherens junction proteins E- and VE-cadherin, leading to cell-cell junctions rupture [160]. The observed leakage of lactate dehydrogenase (LDH) indicates moreover the impending death of the infected host cells [2,114,160,162].

Exl A enables bacteria to proliferate in the lungs and to spread to other organs [159], such as the spleen of mice infected through a pulmonary pathway [157]. Thus, Exl A causes both the disruption of the alveolar-capillary barrier and necrosis of the epithelial and endothelial cells, culminating in hemorrhagic pneumonia, which in turn promotes bacterial dissemination [159]. In addition, Exl A induces the efflux of K^+^ through thereby altering the concentration of cytosolic K^+^ and eliciting activation of the NLRP3/Apoptosis-associated speck like protein containing a caspase recruitment domain (ASC) inflammasome. The resulting caspase-1 activation will cause the release of IL-1β [2]. Exl A can also induce inflammatory pyroptotic death of macrophages through the activation of the NLRP3 inflammasome [114].

### 3.6. Alkaline Protease (AP)

AP is a 50 kDa Zn^2+^-metalloprotease exotoxin expressed by the type I secretion system (T1SS) of *P. aeruginosa* [163,164,165] associated with keratitis, otitis media, CF, and bacteremia [166,167,168,169] that can lead to potentially fatal outcomes in patients with CF, as well as in immunocompromised individuals [165,170]. AP activates the epithelial sodium channel (ENaC) in CF patients, largely due to cleavage and maturation of the γ subunit [169]. Activation of ENaC decreases airway surface liquid volume and as such decreases mucociliary clearance in the lungs of CF patients, which facilitates *Pseudomonas* colonization [169].

### 3.7. Lipopolysaccharide (LPS) from P. aeruginosa

In general, G^−^ bacterial endotoxin or LPS can exert its functions either extra- or intracellularly. In response to extracellular LPS, toll-like receptor 4 (TRL4) induces both a transcriptional innate and adaptive immune response, characterized by increased cytokine expression and Nod-like Receptor (NLR)-mediated canonical inflammasome activation [171,172,173]. In the alveolar space, LPS may provoke the release of inflammatory mediators in resident cells, including TNF, IL-1β, and IL-6 [174]. The TLR4-MD-2 signaling complex can distinguish LPS chemotypes (smooth versus rough) and functions in a MyD88-dependent or a CD14, MyD88-independent manner [175]. The MyD88-dependent, but not the MyD88-independent pathway of TLR4 signaling was shown to be crucial in clearing non-typeable *Haemophilus influenzae* from the mouse lung [176]. LPS can moreover stimulate granulocytes to release neutrophil extracellular traps (NETs) in a process termed NETosis, albeit not very potently.

LPS provokes alveolar-interstitial and interstitial-endothelial barrier breakdown, leading to respiratory failure [38,177]. Intracellular LPS from, e.g., *Escherichia coli*, *Salmonella typhimurium*, *Shigella flexneri,* and *Burkholderia thailandensis* can activate mouse caspase 11 to cause pyroptotic cell death and IL-1β processing in a gasdermin D-dependent manner [178,179,180]. In human cells, the orthologs of mouse caspase-11, i.e., caspases 4 and 5, can potentially cause similar responses [172]. As such, LPS can affect both the innate (TLR4) and the adaptive immune system, the latter upon intracellular LPS-mediated IL-1β and IL-18 release, since these IL-1 family members are involved in both innate and adaptive immunity [173].

LPS from *P. aeruginosa* (LPS_PA_) is produced in both smooth and rough forms to protect the bacterium against host defense mechanisms. It enables *P. aeruginosa* to live in different ecological niches and to establish an infection there [181]. More importantly, it is associated with the pathogenesis of *P. aeruginosa* during COPD, CF, and pneumonia [34,35,38]. In alveolar epithelial cells, LPS_PA_ regulates airway epithelial ion transport at least by three ways [182,183,184]. Firstly, it can induce the release of ATP from alveolar type 2 (AT2) cells leading to activation of the phospholipase C-Protein kinase C (PLC-PKC) pathway through stimulation of the P2Y2 purinergic receptors. Ultimately, this activation cascade decreases the surface expression and activity of ENaC [182]. Secondly, LPS_PA_ activates ERK1/2 and p38 MAPK pathways, which attenuate ENaC-α mRNA expression by reducing ENaC-α promoter activity [183]. Thirdly, LPS_PA_ provokes Ca^2+^ release from thapsigargin-sensitive stores and Ca^2+^ entry by both transient receptor potential (TRP) and L-type calcium channels with subsequent stimulation of Cl^−^ secretion by the CFTR channel in human bronchial epithelium cells [184]. These actions arise within a few minutes and could be considered the first perceived signals by the epithelial cells during *P. aeruginosa* infection [184]. Accordingly, in CF patients, due to the lack of functional CFTR, the pro-secretory effect of LPS on Cl- transport would be expected to be muted and might assist the persistence of infection by reduced mucociliary clearance [184]. Indeed, in the normal lung, mucociliary clearance is increased by *P. aeruginosa* as a defense strategy [184].

In addition, LPS_PA_ can modulate mucin overproduction in chronic inflammatory airway diseases [34]. LPS_PA_ induces the production of reactive oxygen species (ROS) in human airway epithelial cells [34] through protein kinase C (PKC)-NADPH oxidase signaling pathways. These events in turn foster the release of transforming growth factor-α (TGF-α), which upregulates expression of the gel-forming mucin MUC5AC [34]. MUC5AC is generally considered a major airway mucin, produced by goblet cells in the tracheobronchial surface epithelium, and is highly expressed, not only in human bronchial epithelium, but also in bronchial submucosal glands. It has the typical viscoelastic property of mucin that is needed for clearance and maintenance of integrity of the epithelium in order to protect from dehydration and from potential pathogens [185]. As such, upregulation of MUC5AC is an important factor associated with morbidity and mortality of patients with asthma, COPD and CF [34,186,187]. In vitro and in vivo data further show that LPS_PA_ induces the production of ROS in human airway epithelial cells [34], enhances paracellular permeability of airway epithelium, and significantly increases lung inflammation [188]. During infection by *P. aeruginosa*, LPS_PA_ activates mast cells, which decrease the levels of claudin-1 and occludin gene expression that results in pulmonary permeability [189,190].

## 4. *Salmonella enterica*

*S. enterica* is a Gram-negative, motile rod-shaped facultative intracellular pathogenic bacterium [191,192]. Although *Salmonella* species are mainly recognized to cause foodborne disease [193] and are not considered as typical respiratory pathogens, their LPS may nevertheless cause significant perturbations in lung function [194,195].

### LPS of S. enterica (LPS_SE_)

Intratracheal aerosolization of LPS_SE_ in rats resulted in ARDS, characterized by neutrophilic alveolitis, capillary endothelial damage, platelet sequestration, and pulmonary edema [196].

## 5. *Escherichia coli*

*E. coli* is a Gram-negative, rod-shaped, facultative anaerobic bacterium [197]. It is ubiquitous in the human gastrointestinal tract [198]. Pulmonary infections by *E. coli* are relatively rare [199], but can lead to *E. coli* pneumonia both in the community and in the hospital [200]. LPS_SE_ is, however, the main inducer of sepsis-related ARDS. As such, many studies have used LPS from *E. coli* to elucidate the details of endotoxin-induced lung injury [201,202,203,204,205,206,207,208,209,210,211,212,213,214,215,216,217,218].

### 5.1. LPS of E. coli (LPS_EC_)

LPS_EC_ can contribute to the pathogenesis of ALI and ARDS [37,188,189,190], characterized by infiltration of neutrophils and macrophages, the release of pro-inflammatory mediators such as IL-1β, IL-6, IL-8/CXCL8, IL-18, IL-23, TNF, and MIP-2, the decreased release of anti-inflammatory cytokines such as IL-4 and IL-10, and the disruption of pulmonary alveolar epithelial-capillary barrier integrity [204,206,207,208,209,210,211]. In the respiratory epithelium, LPS_EC_ can modulate α-ENaC expression [212], damage bronchiolar and alveolar epithelial cells [201], stimulate AT1 cells to produce a number of pro-inflammatory mediators [213], and increase expression of a calcium-activated chloride channel (CLCA1) [214].

LPS_EC_ causes a pulmonary vascular leakage, and endothelial cell apoptosis, as well as cell contraction, actin reorganization and, thus, loss of the endothelial barrier integrity [201,202,203,215,216,217]. However, in PMNs, LPS was shown to rather blunt apoptosis [218]. LPS downregulates SOX18, which is a barrier-protective protein, which further increases the vascular leak [219]. Moreover, LPS_EC_ can cause PMN-dependent lung injury in rabbits through the recruitment and activation of PMNs, with the chemokine IL-8/CXCL8 playing an important role in this biological action [207].

### 5.2. Exotoxins of E. coli

Apart from its endotoxin (LPS_EC_), *E. coli* also expresses several exotoxins. These include, among others, Hemolysin (HlyA), cytotoxic necrotizing factor-1 (CNF-1), and vacuolating autotransporter toxin (Vat) (reviewed in [220]). With the exception of HlyA, the relevance of these exotoxins for lung injury associated with *E. coli* infections in man remains rather understudied. HlyA is a 110-kD pore-forming exotoxin that has been implicated in the development of acute lung injury [221]. In particular, AMs are sensitive targets for HlyA attack, and respond with a marked pro-inflammatory lipid mediator synthesis. HlyA is considered the prototypical type I secretion protein in G^−^ bacteria. All repeats-in-toxin (RTX) family members share a motif of nine amino acid repeats near the C-terminus involved in Ca^2+^-binding. Unlike the homologous RTX leukotoxins, *E. coli* HlyA is cytotoxic to many different host cell types and towards many species of hosts [222]. CNF1 is a 115-kD protein catalyzing the deamidation of a conserved glutamine residue causing the activation of three members of the Rho family of GTP-binding proteins, i.e., RhoA, Cdc42, and Rac. This causes cytoskeletal rearrangements, cell cycle disruption, and interruption of signaling pathways [223]. CNF1 treatment of PMNs increased their production of ROS but blunted their ability to phagocytize bacteria [224]. Vacuolating AT toxin (Vat) has been demonstrated to be a significant virulence factor in avian pathogenic *E. coli* using respiratory and cellulitis infection models of disease in broiler chickens [225].

## 6. *Bordetella pertussis*

*B. pertussis* is a Gram-negative, strictly aerobic, encapsulated, non-spore forming coccobacillus [226]. This microorganism is highly contagious and causes acute respiratory illness through person- to-person transmission by aerosolized respiratory droplets. It adheres to ciliated cells of the upper and lower respiratory tract to establish colonization using its numerous virulence factors including its exotoxins [227]. *B. pertussis* causes severe paroxysmal coughing, known as whooping cough, in adults and infants [227,228,229], sometimes also accompanied by pneumonia or otitis media.

### 6.1. Pertussis Toxin (PTX)

Pertussis toxin (PTX) is a 105 kDa [230] multi-subunit exotoxin secreted across the bacterial outer membrane by a type IV secretion system, i.e., a transport platform used by *B. pertussis* to secrete its virulence factors in order to invade the host cells as the first-line adhesion factor [227,231,232,233]. The PTS1 subunit of PTX is an adenosine diphosphate (ADP)-ribosyltransferase that inactivates the alpha subunit of heterotrimeric G_i_/_o_ proteins [234]. PTX plays a major role in the pathogenesis of infants’ pertussis, modulation of immune and inflammatory response of the infected host and paroxysmal cough of pertussis [234]. PTX promotes bacterial dissemination beyond the initial site of infection and in severe infant pertussis; PTX mediates leukocytosis as well as pulmonary hypertension through impacting other organs outside the respiratory system [230,234]. PTX facilitates colonization of the respiratory tract by *B. pertussis*, exacerbates and prolongs airway inflammatory responses and inhibits the resolution of inflammation [230,235,236]. In vitro and in vivo studies have further demonstrated that PTX reduces pulmonary barrier function [237,238]. It increases PKC-mediated endothelial permeability in pulmonary artery endothelial cells in vitro [239,240,241]. PTX signals through G protein-coupled receptors (GPCRs), which are involved in the biogenesis and maintenance of epithelial tight junctions. As such, pulmonary PTX instillation leads to the formation of pulmonary edema in mice [230,240,242].

In the early phase of infection, PTX inhibits neutrophil recruitment to the respiratory tracts [243,244], whereas in later phases, PTX aggravates neutrophil recruitment into the alveolar space through the increased production of neutrophil-attracting chemokines including CXCL1, CXCL2, CXCL5, and IL-17A by AMs [245]. By targeting resident airway macrophages, PTX inhibits their anti-bacterial activity [246], but the exact mechanisms for suppression of AM activity remains to be further investigated [247]. PTX can cause -or at least represents a co-factor of- pulmonary hypertension through inhibition of GPCRs in the heart and in the lungs during severe pertussis infection [247]. PTX acts on inflammation together with its other physiological actions that are involved in the pathology of coughing [230]. Accordingly, therapeutic targets of PTX activity can be considered beneficial in order to decrease the severity of the patient’s’ cough during infection [230].

### 6.2. Adenylate Cyclase Toxin (ACT)

Adenylate Cyclase Toxin (ACT) is a pore-forming exotoxin of 200 kDa [248] that is a member of the repeats-in-toxin (RTX) family of proteins, secreted by the type I secretion system by all virulent strains of *B. pertussis* [249,250]. It is also a calmodulin-activated adenylate cyclase enzyme, a multifunctional molecule bearing both catalytic and toxic capabilities that occupies a critical role in pathogenesis of *B. pertussis* through its capability to disarm immune cells and manipulate cellular signaling in host cells [251,252,253]. Considering the complementary action of PTX and ACT in the pathogenesis of infection, PTX is crucial for the initial establishment of infection, whereas ACT plays its role during bacterial persistence in the respiratory tract [254]. In humans, ACT and PTX can cooperatively contribute to pathogenesis of *B. pertussis* infection by establishing the respiratory disease pertussis or whooping cough [230]. To penetrate the host cell, ACT binds to the target cell membrane by its C-terminal portion and delivers its catalytic moiety into the cytosol [255]. Nevertheless, the main passage by which ACT can enter host cells is delivery through outer membrane vesicles [256]. After this membrane translocation, calmodulin activates the catalytic domain of the toxin and enables it to convert cellular ATP into cyclic adenosine monophosphate in phagocytic immune cells, such as neutrophils and macrophages [257,258,259]. The elevated intracellular cAMP may inhibit phagocytosis [232]. ACT can also inhibit chemotaxis of macrophages and can induce apoptosis [260,261,262]. In addition, ACT forms lytic cation-selective pores in the plasma membrane of target cells, as such, contributing to the perturbation of ion homeostasis, launching signaling pathways and promoting cell lysis [263]. Pores formed by ACT are size-tunable and comprise heterogeneous architectures such as lines, arcs, and rings [264]. Depending on the incubation time and toxin concentration, the size of pores evolves differently to provide a channel for the flux of solutes containing large molecular mass whereas vesicle integrity is retained [264].

## 7. *Bacillus anthracis*

*B. anthracis* is a Gram-positive/variable encapsulated, rectangular rod-shaped bacterium, capable of forming endospores, belonging to the genus *Bacillus* and family Bacillaceae [265]. It is an aerobic or facultative-anaerobic bacterium that can live everywhere in the environment [266]. By utilizing its virulence factors, including an anti-phagocytic polyglutamic capsule and the anthrax toxins [267], *B. anthracis* infects animals, especially herbivores and humans [266].

### Anthrax Toxins

Anthrax toxin is a tripartite AB toxin consisting of three soluble non-toxic proteins released by *B. anthracis* [268,269,270]. These proteins are i) protective antigen (PA) with a molecular weight of the 83 kDa, ii) the lethal factor (LF), which is a Zn^2+^-dependent metalloprotease with a molecular mass of ∼90 kDa and iii) edema factor (EF) which is a Ca^2+^/calmodulin-dependent adenylate cyclase, with a molecular mass around 90 kDa [267,271,272]. The PA subunit binds to the extracellular von Willebrand factor A domain of the cellular Anthrax Toxin Receptor (ATR), a type I membrane protein [273], while the subunits LF and EF have toxic enzymatic functions [267,271]. Accordingly, assembly of LF and PA makes Anthrax lethal toxin (LeTx), whereas assembly of EF and PA forms Anthrax edema toxin (EdTx) [274]. Edema factor (EF) is an adenylate cyclase that increases endothelial and epithelial monolayer permeability and impairs host defenses through a variety of mechanisms including inhibition of phagocytosis; lethal factor (LF) is a zinc-dependent protease that cleaves mitogen-activated protein kinase kinase and causes lysis of macrophages. This helps the bacterium evade the immune system and to kill the host during a systemic infection. 

Inhalation anthrax is the most severe form of anthrax [275], with early symptoms similar to a cold or the flu, including shortness of breath, mild chest discomfort, and nausea. However, ultimately potentially lethal severe respiratory failure caused by hemorrhagic mediastinitis and pulmonary edema can arise [275]. The pathophysiology of inhalational anthrax is determined by the interaction between two toxins of *B. anthracis* and a capsule interacting with various cell partners in host organs [276,277]. This process starts with the inhalation of *B. anthracis*’ spores [278]. The lungs act on the one hand as a portal to disseminate spores into the deeper lying organs. On the other hand, the bacteria or spores have a local impact on pulmonary compartments [278,279,280,281,282,283,284]. In both cases, anthrax toxins play important roles in the establishment of the infection.

Regarding the dissemination through the lungs, so far, three models of inhalational *B. anthracis* dissemination have been described. First, spores are transported with an intermediate intracellular step, such as phagocytosis by AMs, dendritic cells or monocytes and are then transported to the regional draining lymph nodes [278,279,280]. Secondly, spores germinate in the lumen of the airways and start to produce exotoxins and proteases, which cause disruption of the alveolar endothelial-to-epithelial barrier and opens up a passage for vegetative bacteria to escape through the disrupted barrier, e.g., in lymphatics, injured vessels or vessel walls towards the regional draining lymph nodes [281]. Thirdly, without affecting the integrity of epithelial cells and through adherence to and internalization by polarized lung epithelial cells, spores can pass through the cells and exit from the basolateral side of pneumocytes [283]. In all these three paths, spores or germinated bacteria travel to the local lymph nodes after crossing the barrier, germinate, replicate, and release toxins, which results in hemorrhagic mediastinitis [284,285]. Subsequently, bacteria can enter into the bloodstream and continue proliferating, being systemically disseminated to nearly all organs including lungs [284]. The toxins can locally pervade neighboring cells, such as pulmonary vascular endothelial cells and thereby cause pulmonary and peripheral edema [285].

Regarding the impact of toxins directly in the lungs, inhaled spores germinate apically at a mucus-secreting air-liquid interface pulmonary barrier and produce vegetative forms of bacteria with the ability to produce and release exotoxins as essential factors for the anthrax pathogenesis [286,287]. To review the pathogenicity of anthrax toxins to lungs at the cellular level, it may be more appropriate to discuss each of the toxins (LeTx and EdTx) separately. Accordingly, it has been shown in a model of isolated perfused rat lungs that they have different impacts on pulmonary vascular pressures and permeability [288]. However, in vivo studies revealed that these toxin components may cooperate synergistically to cause edema formation and cell death. Thus, the concerted action of the two should not be overlooked in practice [289]. It has been shown in vitro and in vivo that LeTx creates most characteristics of an anthrax infection including cytokine-independent vascular dysregulation and collapse, pulmonary vascular constriction, disruption of the rat pulmonary microvascular endothelial barrier integrity leading to severe blood vessel leakage, pulmonary edema, and disrupted gas exchange that clinically leads to ALI/ARDS [275,288,290,291,292,293,294].

LeTx can also target alveolar epithelial cells, as they provide the receptor proteins, needed for the binding of LeTx [295]. LeTx has been shown to provoke actin-rearrangement and to destroy the formation of desmosomes, which results in the impairment of barrier function and in the reduction of the epithelial surfactant production [295,296]. LeTx, moreover, inhibited the secretion of KC (the mouse functional orthologue of human IL-8/CXCL8), IL-6, and MIP-2 [297]. EdTx also indirectly affects endothelial cells by manipulating the release of inflammatory mediators [298]. It enhances central actin stress fibers and changes the distribution of VE-cadherin [299]. Noteworthy is that human AMs are resistant to the immunosuppressive effects of LeTx, which is probably the reason for the lack of detection of the pathogen in the alveolar surface in animal models as well as in autopsy studies [300].

## 8. *Listeria monocytogenes*

*L. monocytogenes* is a Gram-positive rod-shaped motile intracellular pathogenic bacterium [301]. It is a facultative anaerobic bacterium that is ubiquitously distributed in the environment [302,303]. *L. monocytogenes* is capable of overpassing four physiological barriers including the intestinal boundary and blood-brain boundaries, as well as the fetoplacental and alveolar-capillary barriers [21,304]. *L. monocytogenes* mainly infects immunocompromised and to a fewer extent also immunocompetent individuals, including pregnant women, neonates, the elderly, and debilitated patients, causing gastroenteritis, central nervous infections including meningoencephalitis, cerebritis or rhombencephalitis, abortion, septicemia and pneumonia [302,304,305,306,307,308,309,310]. It should be noted that pneumonia induction by *L. monocytogenes* is an event, which predominantly will occur in immune-compromised individuals.

### Listeriolysin O (LLO)

The listerial toxin listeriolysin-O (LLO) is 59 kDa [304] thiol-activated pH-regulated cholesterol-dependent cytolysin and pore-forming exotoxin [304,311] that can form transmembrane β-barrel pores with a diameter of up to 35 nm [304,312] in the plasma membrane of host cells [18]. Regardless of the destructive nature of membrane perforation, this pore formation provides a passage for the alteration of ion gradient such as influx of Ca^2+^ into host cells [16,313,314].

LLO causes a dysfunction in the ENaC channel in airway epithelial cells, especially by targeting the crucial α subunit [21,315]. ENaC-α is not only crucial for promoting Na^+^ reabsorption across Na^+^-transporting epithelia but also for its role in strengthening capillary barrier function in lung microvascular endothelial cells [315,316]. Thus, targeting ENaC-α expression impairs structural and functional properties of ENaC and weakens the capillary barrier function [9,316]. LLO decreases the expression of ENaC-α at least partially through disabling positive regulators of ENaC including serum and glucocorticoid-dependent kinase (Sgk-1) and protein kinase B (Akt-1). This in turn decreases the surface expression and of ENaC- α (as shown in the human bronchiolar epithelial H441 cell line) and thus the capacity of ENaC to take up Na^+^ [21]. In addition, the LLO-induced increase in intracellular Ca^2^ activates protein kinase C-alpha (PKC-α), which in turn induces capillary leak. As such, LLO launces at least two different mechanisms that promote pulmonary edema formation: capillary leak and impaired liquid clearance [21,316,317,318].

In order to prevent the deleterious actions from LLO on Ca^2+^ influx, a reaction product of the phosphatidylcholine-specific phospholipase C (PC-PLC) of *L. monocytogenes* -phosphocholine (ChoP)- has the capacity to potently inhibit intra- and extracellular activities of LLO and also of PLY, including calcium influx, mitochondrial damage, and apoptosis [319].

## 9. *Streptococcus pneumoniae*

*S. pneumoniae* is a slightly pointed coccus or diplococcus and a capsule-forming Gram-positive pathogenic bacterium. Pneumococci represent the leading cause of bacterial pneumonia and of community-acquired pneumonia worldwide. Due to its high morbidity and mortality, this disease represents one of the most important challenges and urgencies in clinical medicine. *S. pneumoniae* is also a prominent cause of sepsis, otitis media, and meningitis in adults [320,321]. *S. pneumoniae* mostly colonizes in the normal nasopharyngeal flora asymptomatically, but it can cross this niche and migrate to the brain, the blood, and the lower respiratory tract [23,322,323]. *S. pneumoniae* can be carried over from person to person via droplets/aerosols [324]. Among the many colonization and virulence factors that *S. pneumoniae* possesses, the pore-forming toxin pneumolysin (PLY) plays a prominent role in the pathogenesis during lung infection [325].

### Pneumolysin (PLY)

PLY is a thiol-activated multifunctional pore-forming exotoxin with a molecular weight of 53 kDa [326,327]. PLY binds free cholesterol and inserts itself into the lipid-rich bilayer of the cell membrane, where it assembles into a ring containing 30-50 monomers with a diameter of about 35 nm [328,329]. Since these pores are Ca^2+^ permeable, they promote an increase of intracellular Ca^2+^ levels [11,15]. PLY is not actively secreted into the extracellular space [330] but is released as a result of *S. pneumoniae* autolysis [9,331] or upon antibiotic treatment [332]. Once released, PLY can induce profound capillary leak, as such aggravating pulmonary permeability edema associated with pneumonia [11,322,332], ALI and ARDS [333,334]. PLY can moreover cause acute exacerbation of idiopathic lung fibrosis (IPF).

**Figure 1:**
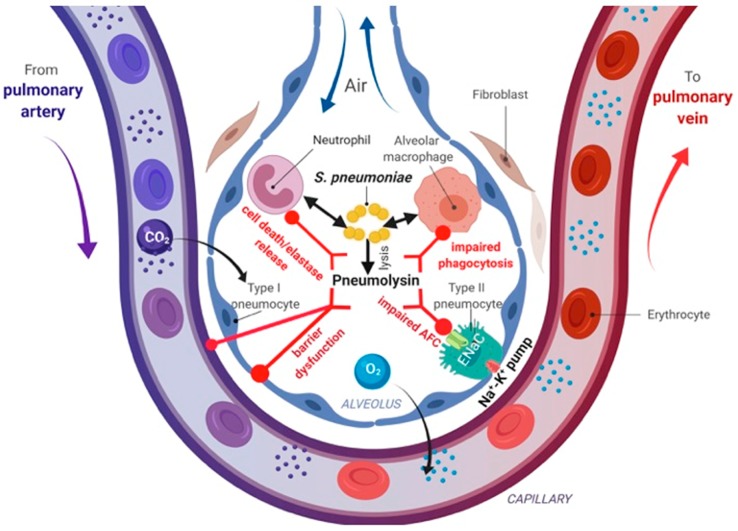
Multiple actions of pneumolysin (PLY) fostering acute lung injury during *S. pneumoniae*-induced pneumonia. Autolysis or antibiotics-induced lysis of the pathogen causes the release of the pore-forming toxin PLY from *S. pneumoniae*. PLY induces chemokine production in alveolar epithelial cells, fostering the infiltration of neutrophils and non-resident alveolar macrophages (AMs) in the alveolar space. PLY induces neutrophil lysis and the release of elastase, which in turn impairs phagocytosis capacity of AMs. Together these actions will impair immune defense against *S. pneumoniae*. PLY furthermore impairs both the alveolar epithelial and capillary barriers through the disruption of adherens and tight junction proteins and through lytic effects in AT1/2 and microvascular endothelial cells. Moreover, PLY impairs Na^+^ uptake in AT1/2 cells, which mediates alveolar fluid clearance, at least partially through the induction of ENaC dysfunction.

In view of its wide range of deleterious activities contributing to ALI, we have summarized PLY’s actions in the lungs in Figure 1. In the alveolar space, pneumococcal enzyme LytA or autolysin mediate autolysis of the bacteria thus inducing the release of PLY [321]. In the presence of antibiotics, this release can lead to high alveolar concentrations of the toxin [331]. When both neutrophils and PLY are present in the alveolar space, PLY will form pores in the neutrophil membrane, which in turn induces cell death and/or the release of neutrophil elastase (NE) (Figure 1) [321]. Subsequently, neutrophil elastase will impair macrophage phagocytic activity and will induce detachment and death of alveolar epithelial cells and the generation of the neutrophil attractant chemokine IL-8/CXCL8 [321]. In addition to the above-mentioned impact of PLY on AMs, the toxin can also induce necroptosis in these cells during acute bacterial pneumonia [5]. PLY induces Ca^2+^-dependent increase of prostaglandin E2 and leukotriene B4 by both resting and chemoattractant-activated human neutrophils in vitro [335]. In addition, the pore-forming toxin can increase PAF and thromboxane A2 synthesis by these cells [336]. Apart from promoting IL-8 generation, PLY also fosters a hepoxilin A3-dependent neutrophil recruitment across the pulmonary epithelium in a pore-dependent fashion [337].

Through its cytotoxic, barrier-disruptive, and complement-activating properties, PLY participates in the pathogenesis of ALI and promotes bacteremia [332]. PLY’s lytic pores may cause alveolar hemorrhage and dissemination of alveolar microorganisms into the bloodstream [338,339,340,341]. PLY has also cytotoxic properties, as it can causes caspase 6-dependent apoptosis in lung epithelium and capillary endothelium [338,339]. Interestingly, different immune cells were shown to exert differential sensitivity to the pore-forming-induced cytotoxic activities. As such, T cells were shown to be highly susceptible, whereas alveolar macrophages are more resistant [342]. Plasma membrane damage caused by cytolysin-induced pore formation can however be repaired through a mechanism involving Ca^2+^-activated lipid scramblase TMEM16F, which in turn promotes repair by enhancing membrane fluidity and the release of extracellular vesicles carrying damaged membranes [343].

PLY-induced damage in lung tissue can be sensed by the NOD-, LRR-, and pyrin domain-containing protein 3 (NLRP3) inflammasome [344]. However, different pneumococcal serotypes can activate these innate immune receptors to a different extent and some, such as serotype 1 and 8 isolates can evade sensing by inflammasomes altogether [344]. NLRP3 protects the alveolar barrier during pneumococcal PLY-induced ALI by preventing detachment of epithelial cells [345]. PLY activates the NLRP3 inflammasome to promote generation of pro-inflammatory cytokines independently of TLR4 [344,346].

PLY’s role in inflammation is highly complex, since the toxin can induce both pro- and anti-inflammatory actions. PLY can promote inflammation by means of activating the NF-κB and p38 MAP kinase pathways [11,347,348]. Moreover, in view of its capacity to increase intracellular Ca^2+^ levels, PLY activates Ca^2+^-dependent enzymes, including PKC-α [11], which in turn causes inhibitory phosphorylation of endothelial nitric oxide synthase (eNOS) and barrier dysfunction [321,339,349]. By contrast, PLY binding to the mannose receptor C type 1 (MRC-1) in dendritic cells and AMs was recently shown to blunt pro-inflammatory cytokine responses and toll-like receptor signaling and to upregulate the cytokine suppressor SOCS1, as such, facilitating pneumococcal internalization into non-lysosomal compartments [350]. Increased intracellular Ca^2+^ provoked by PLY’s pore forming actions moreover induces pathways targeting mechanisms mediating alveolar liquid clearance. This predominantly includes vectorial Na^+^ uptake by the ENaC in AT1/2 cells [9] (Figure 1).

## 10. Conclusions

Healthy humans are colonized and co-exist with over 100 trillion bacteria as well as significant numbers of viruses, fungi, and archaea [351]. These microorganisms, thus, outnumber human cells by about 10–100-fold and constantly interact with the surrounding environment. Microbial infection is highly relevant to the biological functioning of the respiratory system and, thus, to disease pathogenesis in the host. The immune system is crucial to protect the lung from infection, as evidenced with, e.g., severe neutropenia or acquired immune deficiency syndrome, where pneumonia frequently occurs and is by far the most frequent infection. Repeated or non-resolving infections may be key events in pathogenic evolution of chronic pulmonary diseases, such as COPD, CF lung disease, bronchiectasis, and others. The biological system includes local and systemic inflammation and immune response and their reparative control mechanisms. During the dynamic interaction with the environment, lungs are highly influenced by inhalational stressors, such as cigarette smoke, ozone and occupational toxic agents, as well as by host factors, including the nutrition-, sleep-, and exercise-dependent performance of our immune defense system. This is further influenced by infectious insults, as documented by the high susceptibility for severe bacterial pneumonia during the course of influenza virus infection. Such complications quite frequently lead to respiratory failure or to septic shock. Whereas commensal organisms may provide the host with a number of benefits (e.g., contributing to metabolism or shaping immune competence), the vast majority of infections are caused by organisms that are part of the normal flora (e.g., *S. aureus, S. pneumoniae,* and *P. aeruginosa*) [351]. Only relatively few infections are due to organisms that are strictly pathogens (e.g., *Neisseria gonorrhoeae*, *Treponema pallidum*, *Mycobacterium tuberculosis*, some non-tuberculous mycobacteria).

In conclusion, bacterial toxins may play a key pathogenic role and are thus of highest relevance in many instances of infection. The site- and situation-dependent role of bacterial toxins and the adaptive interplay with the host are crucial factors for the infective potential of the pathogens. Indeed, the role of bacteria and their toxins seems to be determined by both general and highly specific, time-dependent local host and environmental factors, as well as by the ecological status of the microbiome [352].
